# Diseases of the Nervous System Resulting from Accident and Injury

**Published:** 1906-11

**Authors:** 


**Diseases of the Nervous System resulting from Accident and Injury.** By **Pearce Bailey,** A.M., M.D., Clinical Lecturer in Neurology, Columbia University, New York; Consulting neurologist to Roosevelt, Saint Luke's, and Manhattan State Hospitals. Octavo, 628 pages. New York and London: D. Appleton and Company. 1906. (Price: Cloth, ?????).

The author has rewritten and revised his former work on this subject and the result is a book which by reason of its completeness will prove of value to surgeons and physicians, irrespective of their specialties. After considering the organic effects of injury to the nervous system about which there can be no controversy, he passes to the discussion of the late effects of injury.

Trauma as a factor in the causation of paresis and locomotor ataxia receives such judicial treatment that it deserves special notice. Can a person who has never had syphilis or marked hereditary predisposition develop general paresis as the direct consequence of injury to the head? In answer to this he quotes Pettit of the Manhattan State Hospital, "from a study of 2,000 clinical histories, he is forced to believe that head injury is not to be regarded as a sole cause of the disease." He sums up after a review of the views of a large number of the cases reported the world over, that in his opinion "the conclusion is unavoidable that if ever trauma is the sole cause of general paresis, such a causal relationship is extremely unusual and difficult of proof, and is to be accepted only after scrupulous inquiries have eliminated all the many opportunities for error."

The evidence of the traumatic origin of tabes is the Scotch verdict, "not proven." We consider that as a reference book and guide in the medico-legal practice which is liable to come to all physicians, this book will be a great favorite. We know of no book so complete on the subject.

